# Experience of Emergency Department Patients With Using the Talking Pole Device: Prospective Interventional Descriptive Study

**DOI:** 10.2196/mhealth.9676

**Published:** 2018-11-22

**Authors:** Junsang Yoo, Ji Yeong Soh, Wan Hyoung Lee, Dong Kyung Chang, Se Uk Lee, Won Chul Cha

**Affiliations:** 1 Department of Digital Health Samsung Advanced Institute of Health Sciences and Technology Sungkyunkwan University Seoul Republic of Korea; 2 Creative Laboratory Samsung Electronics Suwon Republic of Korea; 3 Department of Gastroenterology Samsung Medical Center Seoul Republic of Korea; 4 Health Information Center Samsung Medical Center Seoul Republic of Korea; 5 Department of Emergency Medicine Seoul National University Hospital Seoul Republic of Korea; 6 Department of Emergency Medicine Samsung Medical Center Sungkyunkwan University School of Medicine Seoul Republic of Korea

**Keywords:** emergency department, health information technology, Internet of Things, mobile phone, patient engagement

## Abstract

**Background:**

Patient engagement is important. However, it can be difficult in emergency departments (EDs).

**Objective:**

The aim of this study was to evaluate the satisfaction of ED patients using a patient-friendly health information technology (HIT) device, the “Talking Pole,” and to assess the factors relevant to their satisfaction.

**Methods:**

This study was conducted in May 2017 at the ED of a tertiary hospital. The “Talking Pole” is a smartphone-based device attached to a intravenous infusion pole with sensors. It is capable of sensing patient movement and fluid dynamics. In addition, it provides clinical information from electronic medical records to patients and serves as a wireless communication tool between patients and nurses. Patients and caregivers who entered the observation room of the ED were selected for the study. The “Talking Pole” devices were provided to all participants, regardless of their need for an intravenous pole upon admittance to the ED. After 2 hours, each participant was given an 18-item questionnaire created for this research, measured on a 5-point Likert scale, regarding their satisfaction with “Talking Pole.”

**Results:**

Among 52 participants recruited, 54% (28/52) were patients and the remaining were caregivers. In total, 38% (20/52) were male participants; the average age was 54.6 (SD 12.9) years, and 63% (33/52) of the participants were oncology patients and their caregivers. The overall satisfaction rate was 4.17 (SD 0.79 ) points. Spearman correlation coefficient showed a strong association of “overall satisfaction” with “comparison to the previous visit” (ρ=.73 ), “perceived benefit” (ρ=.73), “information satisfaction” (ρ=.70), and “efficiency” (ρ=.70).

**Conclusions:**

In this study, we introduced a patient-friendly HIT device, the “Talking Pole.” Its architecture focused on enhancing information delivery, which is regarded as a bottleneck toward achieving patient engagement in EDs. Patient and caregiver satisfaction with the “Talking Pole” was positive in the ED environment. In particular, correlation coefficient results improved our understanding about patients’ satisfaction, HIT devices, and services used in the ED.

## Introduction

The needs of patients and importance of patient engagement are increasing; therefore, informed decision making in emergency departments (EDs) is critical [1,2]. Shared decision intervention, which is based on patients’ proper understanding of their treatment, not only improves patient outcomes but also increases satisfaction and reduces health care utilization [3,4]. One of the important prerequisites for shared decision interventions is that patients have sufficient knowledge about their health care plan [5].

Accomplishing patient engagement in the ED is regarded as difficult because information delivery to patients is disrupted by the hostile and confusing circumstances of the ED [6-8], even if most patients wish to know about their illness and treatment [9-11]. Delivering information is an essential first step in patient engagement [3,4,12]. Unfortunately, the rapid pace of the process and the volume of information required often exceed an individual’s comprehension [1,13]; moreover, interrelated factors, including the uncertainty of diagnosis and treatment, further complicate this situation [14]. However, information transfer does not have to rely solely on the relationship between a patient and their health care provider [15].

Health information technology (HIT) has the potential to improve patient engagement in EDs. The Society for Academic Emergency Medicine has presented strategies to accomplish patient engagement in the ED, including “using HIT to enhance patient communication” [8]. With the systemic constraints of health systems and advancement of the health information technology infrastructure, HIT ranks as the most efficient candidate for improving patient engagement. At present, however, the potential of HIT has not been fully reached owing to deficiencies in design and implementation issues [16,17].

The aims of this study were to evaluate the satisfaction of a patient-friendly HIT device, the “Talking Pole,” in the ED environment and to assess the factors relevant to patient satisfaction.

## Methods

### Introduction to the “Talking Pole”

**Figure 1:**
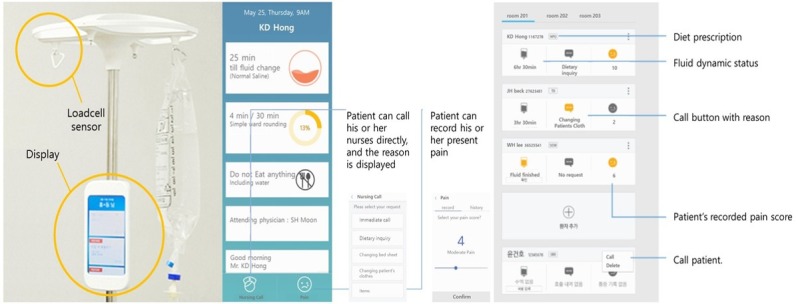
General features of the “Talking Pole” system, from left to right: the “Talking Pole” device, patient display, and dashboard for medical staff.

The “Talking Pole” is a patient-friendly HIT device that was developed to provide smart care to in-patients through the Internet of Things technology. The development team was comprised of both clinical and technical domain experts, and the device consisted of a weight sensor and a smartphone-based display (Figure 1).

The device has the following capacities. First, the device can deliver medical information from the electronic medical records system to the patient. Patients can check their medical schedule, information on medications, vital sign records, diet order information, and so on. Second, the “Talking Pole” enhances communication with medical staff in a subtle way. Two buttons at the bottom of the display, the “Call” and “Pain” buttons, allow patients to enter information for medical staff to see in real-time. Third, the “Talking Pole” checks fluid infusion status in real-time, so patients need not be concerned about receiving the appropriate amount of fluids and nurses can more conveniently monitor the flow. Finally, the “Talking Pole” provides patients with appropriate exercise goals according to their treatment plan and measures actual time spent exercising.

### Study Setting

This is a prospective interventional descriptive study. The study was undertaken at an ED with an annual visit volume of 76,000 in a tertiary academic teaching hospital in Seoul. Participants were recruited from May 1, 2017 to May 31, 2017. The study was approved by the institution’s ethics committee (Institutional Review Board File # SMC 2017-03-034-002).

### Selection of Participants

Inclusion criteria were patients and their caregivers who entered the observation room in the ED and agreed to participate in this study. Usually, patients entering the observation room are distinguished from patients treated in other ED areas in the following ways: those who had finished initial assessment or treatment and those waiting for admission or determined to need monitoring of their condition over time. Such patients receive relatively planned and static treatment. By determining patients with these inclusion criteria, we intended to minimize the extent to which the study affected the subjects’ treatment process. A caregiver was defined as a family member or friend who visited the ED with the patient. The criteria excluded patients declaring a “do not resuscitate”, those younger than 18 years, those whose mental state was not alert, those with a critical medical device, and those at level 1 on the Korean Triage and Acuity Scale (KTAS). The KTAS was developed based on the Canadian Triage and Acuity Scale; level 1 indicates the highest acuity or severity of distress and level 5 indicates the lowest [18]. Patients with higher severity levels were excluded because it was clinically infeasible for them to use the “Talking Pole” device or because would require an amount of information that would exceed the capability of the device.

### Interventions

After obtaining their consent, participants received the “Talking Pole” devices regardless of their need for an intravenous pole and were encouraged to use the device. After 2 hours, each participant was given an 18-item questionnaire developed for this study containing a 5-point Likert scale; questions regarded patients’ satisfaction with the “Talking Pole.” We prepared a total of 5 devices, including 2 extras for use in case of device failure, and the hardware and software of all devices were identical.

### Outcome Measures

The main outcome was the determination of overall patient satisfaction with the “Talking Pole” system. Secondary outcome was a Spearman rank correlation coefficient between overall satisfaction and other questionnaire items. Secondary analysis was performed to determine where to focus and improve in the next iteration of our development process and to identify the factors affecting the users’ satisfaction with HIT devices or services. Identifying these factors is crucial for future researchers and developers to find the best methods of applying HIT services in the clinical setting.

### Data Analysis

Controversy exists among scholars about whether Likert scales should be analyzed in a parametric or nonparametric way. Likert scales are generally considered to be ordinal scales, but they are also used as interval scales [19,20]. In this study, we considered each item of the questionnaire on an interval scale.

We performed subgroup analyses of overall satisfaction and reported the means and SDs. We also examined Spearman rank correlation coefficient to examine associations between overall satisfaction and other factors. Correlation coefficients of |0.7-1| are considered to be strong, |0.5-0.7| are moderate, and less than |0.5| indicate weak relationships [21]. R version 3.4.3 was used for statistical analysis [22].

## Results

### Characteristics of Study Participants

A total of 52 participants were recruited, and there were no cases of dropout. The general characteristics of participants are presented in Table 1.

### Evaluation Outcomes

**Figure 2:**
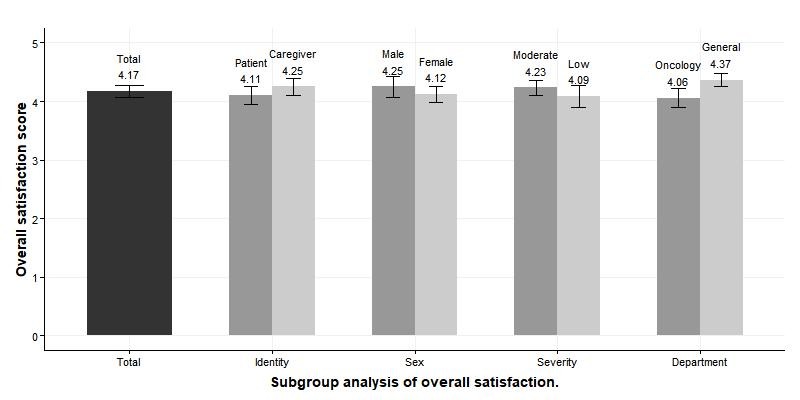
Overall satisfaction score and subgroup scores.

Overall satisfaction with the “Talking Pole” system was measured at 4.17 points. The overall satisfaction score was higher for the male group (4.25 points) than for the female group (4.12 points), the caregiver group (4.25 points) than for the patient group (4.11 points), the moderate-severity group (4.23 points) than for the low-severity group (4.09 points), and the general patients group (4.37 points) than for the oncology patients group (4.06 points), but statistical significance of each subgroup was not verified (Figure 2).

The bars represent SEs of means. “Moderate severity” is indicated by KTAS levels 2 and 3, while “low” includes levels 4 and 5. Under “department,” “general” includes cardiology, gastroenterology, infection, neurology, and nephrology patients.

The mean of participant responses was at least 4.0 points for all items. Items that evaluated interactions with medical staff, such as “call button” and “input pain score,” were rated higher than those that evaluated the information display, such as “display information of username,” “medical staff,” “fluid infusion,” and “dietary prescription” (Table 2).

**Figure 3:**
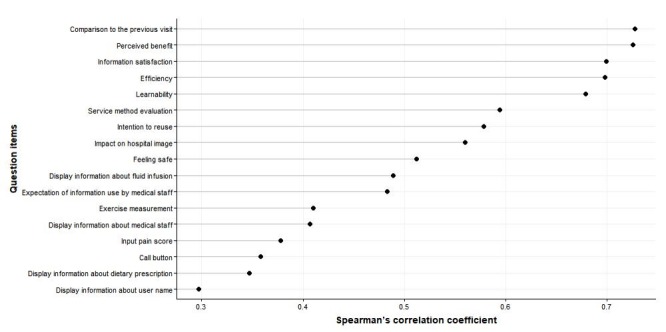
Correlation between overall satisfaction and each question.

Spearman correlation coefficient showed a strong correlation of “overall satisfaction” with “comparison to the previous visit” (ρ=.73), “perceived benefit” (ρ=.73), “information satisfaction” (ρ=.70), and “efficiency” (ρ=.70); on the other hand, items related to function were low (ρ=.29-.48; see Figure 3). All correlation coefficients were significant at *P*=.05.

**Table 1:** General characteristics of study participants.

Characteristics		Patient (n=28)	Caregiver (n=24)	Total (n=52)
**Age (years), mean (SD)**		57.7 (13.9)	50.9 (10.9)	54.6 (12.9)
**Sex, n (%)**				
	Male	16 (57)	4 (17)	20 (38)
	Female	12 (43)	20 (83)	32 (62)
**Diagnosis category, n (%)**				
	Cardiology	1 (4)	0 (0)	1 (2)
	Gastroenterology	2 (7)	1 (4)	3 (6)
	Infectious	5 (18)	4 (17)	9 (17)
	Neurology	1 (4)	1 (4)	2 (4)
	Oncology	17 (61)	16 (67)	33 (63)
	Nephrology	2 (7)	2 (8)	4 (8)
**Severity (Korean Triage and Acuity Scale), n (%)**				
	1	0 (0)	0 (0)	0 (0)
	2	1 (4)	0 (0)	1 (2)
	3	15 (54)	14 (58)	29 (56)
	4	11 (39)	9 (38)	20 (38)
	5	1 (4)	1 (4)	2 (4)

**Table 2:** Mean score for each question

Questions	Score, mean (SD)
Perceived benefit	4.00 (0.74)
Learnability	4.27 (0.69)
Efficiency	4.25 (0.81)
Feeling safe	4.19 (0.86)
Overall satisfaction	4.17 (0.79)
Information satisfaction	4.17 (0.76)
Intention to reuse	4.33 (0.79)
Impact on hospital image	4.38 (0.69)
Comparison to the previous visit	4.38 (0.75)
Display information about user name	4.31 (0.78)
Display information about medical staff	4.25 (0.62)
Display information about fluid infusion	4.27 (0.72)
Display information about dietary prescription	4.38 (0.57)
Call button	4.48 (0.64)
Input pain score	4.56 (0.57)
Exercise measurement	4.46 (0.58)
Expectation of information use by medical staff	4.08 (0.90)
Service method evaluation	4.33 (0.62)

## Discussion

### Principal Findings

In this study, we investigated the overall satisfaction of a patient-friendly HIT device by patients and caregivers in a real clinical environment as well as the correlation between overall satisfaction and other factors based on user surveys. The overall satisfaction score of the “Talking Pole” system was high. In addition, we found a high correlation of overall satisfaction with “comparison to the previous visit,” “perceived benefit,” “information satisfaction,” and “efficiency.” Under the TURF framework, “perceived benefit” and “efficiency” in our survey correspond to “useful” and “learnability” corresponds to “usable” [23]. The findings from the correlation coefficient may be consistent with this framework.

The participants also reported “information satisfaction” and “feeling safe” with scores of 4.17, with high correlation to overall satisfaction, and 4.19, with moderate correlation, respectively. The high satisfaction rate could be partially due to strong informational needs of ED patients. Thus, having personal information transferred directly to them would be satisfying. Based on the study settings and results, we can conclude that it is feasible to use our device to deliver information in a real ED environment.

Sharing information is an essential first step, as well as being a significant barrier, of patient engagement. Prior literature has reported that patients who visit the ED experience severe anxiety and concern due to uncertainty about their disease, diagnosis, treatment, admission, and even medical error [14,24]. Thus, we need to decrease that uncertainty by providing information that patients and caregivers wish to have in a timely and personalized manner. However, until now, satisfying patients’ informational needs has been regarded as difficult, especially in EDs. Delivering information has relied considerably on interpersonal communication between medical staff and patients, but this communication is often disturbed due to a high workload as well as a confusing and complex ED environment [6,7,25].

Under these circumstances, HIT can be a good solution for information delivery as well as for cost and quality of health care [26]. With this in mind, we developed our product, the “Talking Pole,” with the expectation that it would improve patients’ and caregivers’ information-seeking behaviors by delivering medical information directly from the electronic medical records to the patient. However, incorporating HIT into EDs does not guarantee patient engagement. There are numerous unintended consequences associated with imprudent implementation, some even harmful to patients [27-30]. For this reason, we investigated patient and caregiver satisfaction with our devices by testing the “Talking Pole” with ED patients and caregivers in the actual ED environment. Satisfaction, as opposed to other usability factors, could be readily assessed in our study setting. The International Standard for Organization has defined satisfaction as the “extent to which the user’s physical, cognitive, and emotional responses that result from the use of a system, product, or service meet the user’s needs and expectations” [31]. A further explanation states that “a system is satisfying to use if the users have a good subjective impression of how useful, usable, and likable the system is” [23]. Although satisfaction is only one aspect of usability, it is associated with various factors, including intuitive design, ease of learning, efficiency of use, error frequency, and severity [32].

Prior literature has reported that multidisciplinary collaboration involving health care professionals is a factor in the successful application of HIT [33,34], and our experience is consistent with this. In our development process, clinical experts participated in the team from the ideation stage throughout. We thought that this active conjunction between both medical and technical domain experts may help the “Talking Pole” become more feasible by reflecting domain specificity of the clinical environment as well as by uncovering patients’ unmet needs.

Finally, we routinely use the phrase “patient engagement,” but this is an abbreviation of “patient and family engagement” [35]. It is a common phenomenon for a patient to bring a family member, friend, or accompanying person with them when they come to the hospital. Therefore, when we measured the satisfaction of the “Talking Pole,” which is a system designed to improve “patient engagement,” the research team thought it would be appropriate to include caregivers in the participant group.

### Limitations

First, this research was conducted in an ED of a single tertiary academic hospital; readers must be careful when extending their interpretations of these results to other departments or hospitals. However, considering that the need for information is a common phenomenon for patients under a variety of circumstances [25,36-40], the “Talking Pole” has potential applicability to other departments, such as wards. Second, we assessed the satisfaction of the “Talking Pole,” which is only one aspect of usability, so this research cannot conclude that our product is usable. Third, we used a questionnaire that we developed ourselves and that has not been validated; there is a possibility that it contains response biases, such as an acquiescence bias. Further researchers may consider using inversely coded questionnaires to overcome this kind of bias. However, it is not a fundamental solution to this problem, since the acquiescence response style itself tends to produce positive responses regardless of content [41]. Fourth, we did not investigate the patients’ clinical outcomes and usability. Further research is needed to evaluate usability with a validated tool and when the device is implemented in other hospitals or other departments.

### Conclusion

The overall satisfaction of the “Talking Pole” was high, and it highly correlated with “comparison to the previous visit,” “perceived benefit,” “information satisfaction,” and “efficiency.” Through this study, we were able to verify that the “Talking Pole” was able to help meet the needs of patients’ and caregivers’ information-seeking behaviors, which are regarded as the primary barrier of patient engagement in an ED environment.

## Abbreviations

ED: emergency department
HIT: health Information technology
KTAS: Korean Triage and Acuity Scale
